# Protective Effects of Grape Seed Oligomeric Proanthocyanidins in IPEC-J2–*Escherichia coli*/*Salmonella* Typhimurium Co-Culture

**DOI:** 10.3390/antibiotics11010110

**Published:** 2022-01-15

**Authors:** Dóra Kovács, Nikolett Palkovicsné Pézsa, Ákos Jerzsele, Miklós Süth, Orsolya Farkas

**Affiliations:** Department of Pharmacology and Toxicology, University of Veterinary Medicine, 1078 Budapest, Hungary; pezsa.nikolett@univet.hu (N.P.P.); jerzsele.akos@univet.hu (Á.J.); suth.miklos@univet.hu (M.S.); farkas.orsolya@univet.hu (O.F.)

**Keywords:** proanthocyanidins, *Escherichia coli*, *Salmonella* Typhimurium, IPEC-J2, ROS, antioxidant, interleukin, anti-inflammatory, adhesion, barrier integrity

## Abstract

Intestinal epithelium provides the largest barrier protecting mammalian species from harmful external factors; however, it can be severely compromised by the presence of bacteria in the gastrointestinal (GI) tract. Antibiotics have been widely used for the prevention and treatment of GI bacterial infections, leading to antimicrobial resistance in human and veterinary medicine alike. In order to decrease antibiotic usage, natural substances, such as flavonoids, are investigated to be used as antibiotic alternatives. Proanthocyanidins (PAs) are potential candidates for this purpose owing to their various beneficial effects in humans and animals. In this study, protective effects of grape seed oligomeric proanthocyanidins (GSOPs) were tested in IPEC-J2 porcine intestinal epithelial cells infected with *Escherichia coli* and *Salmonella enterica* ser. Typhimurium of swine origin. GSOPs were able to alleviate oxidative stress, inflammation and barrier integrity disruption inflicted by bacteria in the co-culture. Furthermore, GSOPs could decrease the adhesion of both bacteria to IPEC-J2 cells. Based on these observations, GSOPs seem to be promising candidates for the prevention and treatment of gastrointestinal bacterial infections.

## 1. Introduction

Intestinal epithelial cells constitute the largest barrier surface in mammals providing separation from the external environment, especially from dietary antigens and microorganisms [1,2]. Gram-negative bacteria and the endotoxin (lipopolysaccharides, LPS) released from their cell membrane can significantly impair the integrity of the intestinal barrier by causing oxidative stress, inflammation, and morphological damage in epithelial cells [3,4,5,6,7]. Destructions carried out on the intestinal epithelium resulting in the loss of barrier function have been associated with the development of several intestinal and extra-intestinal disorders [2,8]. *Escherichia coli* and *Salmonella enterica* ser. Typhimurium are among the main Gram-negative pathogens causing gastrointestinal (GI) infections in humans and pigs [9,10,11]. Furthermore, the former is commonly associated to the latter as both bacteria are zoonotic, and foodborne transmission with pork products represents a significant proportion of human cases [10,11,12,13]. These bacteria, when isolated from humans, animals and food of animal origin, are frequently resistant to multiple antibiotics [14] and are able to transfer their resistance genes to further microorganisms [11], leading to the spread of antimicrobial resistance along the food chain.

To preserve the health of the GI tract, and to decrease the amount of antimicrobial drugs used for treating intestinal infections, alternative substances are needed in the human and veterinary field, supported by current consumer demands preferring natural, unmedicated products. Flavonoids are natural substances of plant origin, exhibiting beneficial effects on the GI barrier. Even though they are found in several plant-derived foods, to achieve their desired effects, consumption of supplements seems necessary [8]. Proanthocyanidins (PAs) are flavonoids widely investigated for their usage in both human and veterinary medicine due to their various beneficial effects. They have been shown to exhibit antioxidant, anti-inflammatory and anticarcinogenic properties, and activity against different pathogens, including bacteria, viruses, fungi, and parasites [9,15,16,17]. Furthermore, PAs are well known for their ability to inhibit the adhesion of bacteria to cells and other surfaces [18,19,20]. Their protective effect on the cardiovascular [21] and GI [22] systems has also been reported. Among many other fruits, vegetables and seeds, grape seed extract is a rich source of PAs [9,15,17]. Based on their desirable effects, PAs might serve as antibiotic alternatives through the protection of the GI tract against bacterial infections.

IPEC-J2 is a porcine intestinal epithelial cell line, isolated from the jejunum of a neo-natal, unsuckled piglet. IPEC-J2 cells provide a representative model for studying the interaction of bacteria with the porcine intestinal epithelium [23] and effects of food components on the epithelial function [24]. Advantages of this cell line include its non-transformed, non-tumorigenic nature, as well as morphological and functional similarities with in vivo properties of the intestinal epithelium [24]. IPEC-J2 cells are capable of expressing tight junction proteins, as well as synthetizing cytokines, defensins, toll-like receptors, and mucins, and they can be used for studying the antioxidant activity of different substances. The cell line is not only important for investigations targeting the GI health of pigs, but can also serve as a model of the human GI epithelium [25]. Among the non-human cell lines, IPEC-J2 can model human epithelial cells most closely, as the porcine and human GI tracts are similar in many aspects (e.g., size, weight, anatomy, and physiology). As a consequence, IPEC-J2 cells provide a useful model for investigating zoonotic enteric infections that occur in pigs and humans [24].

The aim of this study was to investigate the beneficial effects of grape seed oligomeric pro-anthocyanidins (GSOPs) in IPEC-J2–*E. coli*/*S*. Typhimurium co-culture, as a model for GI infections of humans and pigs.

## 2. Results

### 2.1. Cell Viability Determination

**Figure 1:**
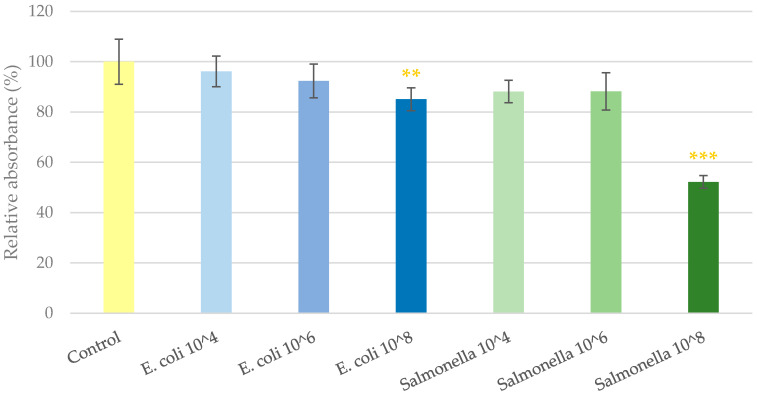
Viability of IPEC-J2 cells after one hour of treatment with bacterial suspensions. Control—treatment with plain medium; *E. coli* 10^4, 10^6, 10^8—treatment with 10^4^, 10^6^ or 10^8^ CFU/mL *E. coli*, respectively; *S*. Typhimurium 10^4, 10^6, 10^8—treatment with 10^4^, 10^6^ or 10^8^ CFU/mL *S*. Typhimurium, respectively. Data are shown as means with standard deviation and expressed as relative absorbance, considering the mean value of control as 100%. *N* = 6/group. Significant difference: ** *p* < 0.01, *** *p* < 0.001, in yellow: compared to the untreated control.

As the first step of this study, the highest tolerable bacterial concentration was determined that could be co-cultured with IPEC-J2 cells unaccompanied by significant cell viability decrease. For this purpose, Neutral Red dye was applied on IPEC-J2 cells after being treated with 10^4^, 10^6^ and 10^8^ colony-forming units (CFU)/mL bacteria for 1 h. The results of the assay can be seen in Figure 1. Bacterial suspensions of *E. coli* and *S*. Typhimurium at the concentration of 10^4^ and 10^6^ CFU/mL did not alter the viability of IPEC-J2 cells. Suspensions of 10^8^ CFU/mL in the case of both bacteria significantly decreased the ratio of viable IPEC-J2 cells in the culture. GSOPs alone did not show a cytotoxic effect on IPEC-J2 cells up to 200 μg/mL for 24 h in previous experiments [9]. Based on these results, and in accordance with the relevant literature [26,27], 10^6^ CFU/mL bacterial suspensions were used in the further experiments.

### 2.2. Intracellular Reactive Oxygen Species Level

**Figure 2:**
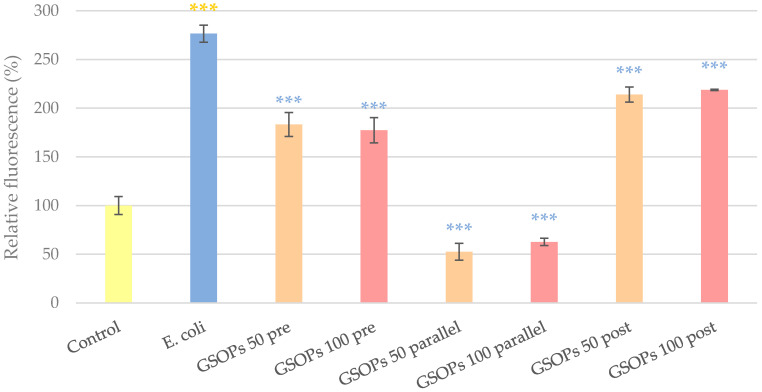
Intracellular reactive oxygen species level of IPEC-J2 cells after one hour of treatment with *Escherichia coli* and grape seed oligomeric proanthocyanidins (GSOPs). Control—treatment with plain medium; *E. coli*—treatment with 10^6^ CFU/mL *E. coli*; GSOPs 50, 100 pre—pre-treatment before *E. coli* infection with 50 and 100 μg/mL GSOPs, respectively; GSOPs 50, 100 parallel—parallel treatment of *E. coli* infection with 50 and 100 μg/mL GSOPs, respectively; GSOPs 50, 100 post—treatment after *E. coli* infection with 50 and 100 μg/mL GSOPs, respectively. Data are shown as means with standard deviation and expressed as relative fluorescence, considering the mean value of control as 100%. *N* = 6/group. Significant difference: *** *p* < 0.001, in yellow: compared to the untreated control, in blue: compared to *E. coli* treatment.

To determine the potential antioxidant effect of GSOPs, changes in the intracellular reactive oxygen species (IC ROS) level of cells were investigated after the addition of bacteria alone and in combination with different GSOP treatments. After 1 h of treatment with 10^6^ CFU/mL *E. coli*, the IC ROS level of cells increased significantly compared to the untreated control, which was significantly alleviated by the administration of GSOPs regardless of the time of GSOPs addition. There was no difference between the effect of GSOPs in lower and higher concentrations (pre-treatment: *p* = 0.88, parallel treatment: *p* = 0.64, post treatment: *p* = 0.93); however, parallel treatment of GSOPs with bacterial infection showed a more pronounced effect than pre- or post-treatment (*p* < 0.001). The results are shown in Figure 2.

**Figure 3:**
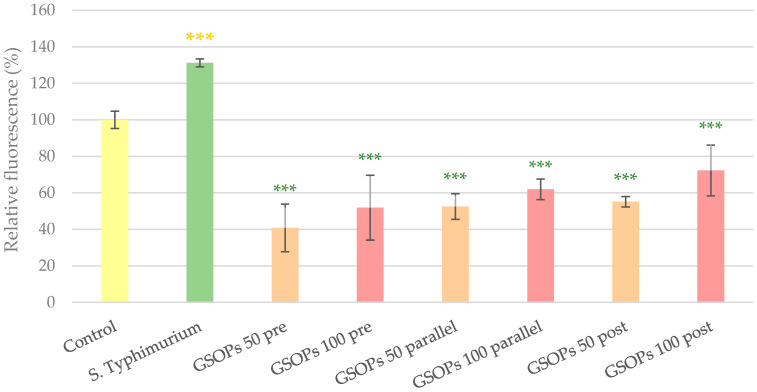
Intracellular reactive oxygen species level of IPEC-J2 cells after one hour of treatment with *Salmonella* Typhimurium and grape seed oligomeric proanthocyanidins (GSOPs). Control—treatment with plain medium; *S*. Typhimurium—treatment with 10^6^ CFU/mL *S*. Typhimurium; GSOPs 50, 100 pre—pre-treatment before *S*. Typhimurium infection with 50 and 100 μg/mL GSOPs, respectively; GSOPs 50, 100 parallel—parallel treatment of *S*. Typhimurium infection with 50 and 100 μg/mL GSOPs, respectively; GSOPs 50, 100 post—treatment after *S*. Typhimurium infection with 50 and 100 μg/mL GSOPs, respectively. Data are shown as means with standard deviation and expressed as relative fluorescence, considering the mean value of control as 100%. *N* = 6/group. Significant difference: *** *p* < 0.001, in yellow: compared to the untreated control, in green: compared to *S*. Typhimurium treatment.

Similar to *E. coli*, application of *S*. Typhimurium on the cells for 1 h resulted in the elevation of IC ROS levels, which was significantly decreased by pre-, parallel and post-treatment with GSOPs. Against *Salmonella*, all treatment types were similarly effective (*p* values between 0.06 and 0.99) and there was no dose-related difference either (pre-treatment: *p* = 0.55, parallel treatment: *p* = 0.72, post treatment: *p* = 0.17) in the activity of GSOPs. The results are presented in Figure 3.

### 2.3. Interleukin Levels

**Figure 4:**
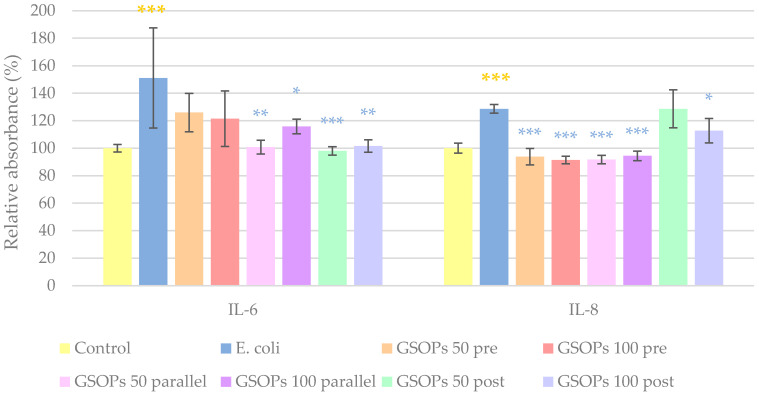
Interleukin-6 (IL-6) and interleukin-8 (IL-8) levels of IPEC-J2 cells after one hour of treatment with *Escherichia coli* and grape seed oligomeric proanthocyanidins (GSOPs). Control—treatment with plain medium; *E. coli*—treatment with 10^6^ CFU/mL *E. coli*; GSOPs 50, 100 pre—pre-treatment before *E. coli* infection with 50 and 100 μg/mL GSOPs, respectively; GSOPs 50, 100 parallel—parallel treatment of *E. coli* infection with 50 and 100 μg/mL GSOPs, respectively; GSOPs 50, 100 post—treatment after *E. coli* infection with 50 and 100 μg/mL GSOPs, respectively. Data are shown as means with standard deviation and expressed as relative absorbance, considering the mean value of control as 100%. *N* = 6/group. Significant difference: * *p* < 0.05, ** *p* < 0.01, *** *p* < 0.001, in yellow: compared to the untreated control, in blue: compared to *E. coli* treatment.

For the evaluation of anti-inflammatory properties of GSOPs, interleukin-6 (IL-6) and interleukin-8 (IL-8) levels of IPEC-J2 cells were measured. Treatment with *E. coli* significantly elevated levels of both IL-6 and IL-8 in the cells. In the case of IL-6, parallel and post-treatments with GSOPs (50 and 100 μg/mL) were able to decrease production of the inflammatory mediator; however, for IL-8, GSOPs pre-treatments also resulted in a significant alleviation of the effect of *E. coli*. Results are demonstrated in Figure 4.

**Figure 5:**
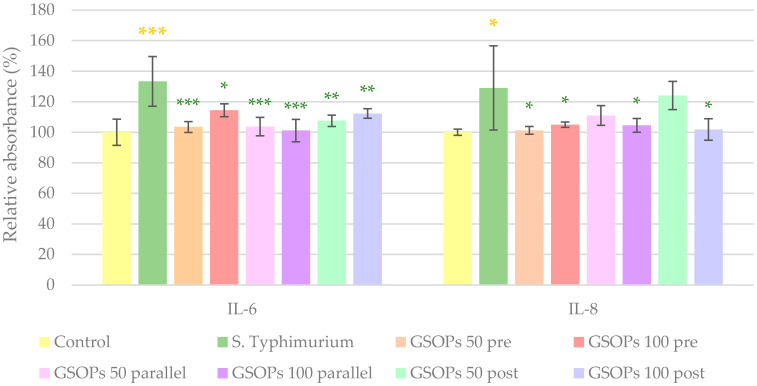
Interleukin-6 (IL-6) and interleukin-8 (IL-8) levels of IPEC-J2 cells after one hour of treatment with *Salmonella* Typhimurium and grape seed oligomeric proanthocyanidins (GSOPs). Control—treatment with plain medium; *S*. Typhimurium—treatment with 10^6^ CFU/mL *S*. Typhimurium; GSOPs 50, 100 pre—pre-treatment before *S*. Typhimurium infection with 50 and 100 μg/mL GSOPs, respectively; GSOPs 50, 100 parallel—parallel treatment of *S*. Typhimurium infection with 50 and 100 μg/mL GSOPs, respectively; GSOPs 50, 100 post—treatment after *S*. Typhimurium infection with 50 and 100 μg/mL GSOPs, respectively. Data are shown as means with standard deviation and expressed as relative absorbance, considering the mean value of control as 100%. *N* = 6/group. Significant difference: * *p* < 0.05, ** *p* < 0.01, *** *p* < 0.001, in yellow: compared to the untreated control, in green: compared to *S*. Typhimurium treatment.

Similar to the effect of *E. coli*, *S*. Typhimurium caused a significant increase in the levels of the investigated cytokines (IL-6 and IL-8). All types and concentrations of GSOP treatments could decrease IL-6 levels, while in the case of IL-8, GSOPs at a 50 μg/mL concentration were not effective when applied before or after bacterial infection. The results can be seen in Figure 5.

### 2.4. Paracellular Permeability

**Figure 6:**
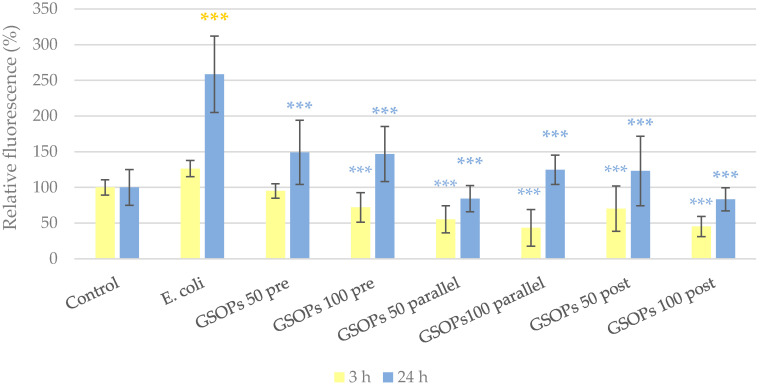
Paracellular permeability of IPEC-J2 cells 3 and 24 h after one hour of treatment with *Escherichia coli* and grape seed oligomeric proanthocyanidins (GSOPs). Control—treatment with plain medium; *E. coli*—treatment with 10^6^ CFU/mL *E. coli*; GSOPs 50, 100 pre—pre-treatment before *E. coli* infection with 50 and 100 μg/mL GSOPs, respectively; GSOPs 50, 100 parallel—parallel treatment of *E. coli* infection with 50 and 100 μg/mL GSOPs, respectively; GSOPs 50, 100 post—treatment after *E. coli* infection with 50 and 100 μg/mL GSOPs, respectively. Data are shown as means with standard deviation and expressed as relative fluorescence, considering the mean value of control as 100%. *N* = 6/group. Significant difference: *** *p* < 0.001, in yellow: compared to the untreated control, in blue: compared to *E. coli* treatment.

The protective effect of GSOPs on the barrier integrity of IPEC-J2 cells was tested via the penetration of a tracer dye through the cell layer. Changes in paracellular permeability were more apparent 24 h after treatment compared to the measurement performed after only 3 h, as the former allowed the tracer dye more time to penetrate. After 24 h, cell layers treated with *E. coli* showed significantly higher paracellular permeability compared to the untreated control, meaning a pronounced destruction of their barrier integrity due to bacterial infection. The deteriorating effect of bacteria was significantly alleviated by the application of GSOPs in all treatment groups and concentrations, and there was no difference between their efficacy (*p* values between 0.18 and 1.00). The results are shown in Figure 6.

**Figure 7:**
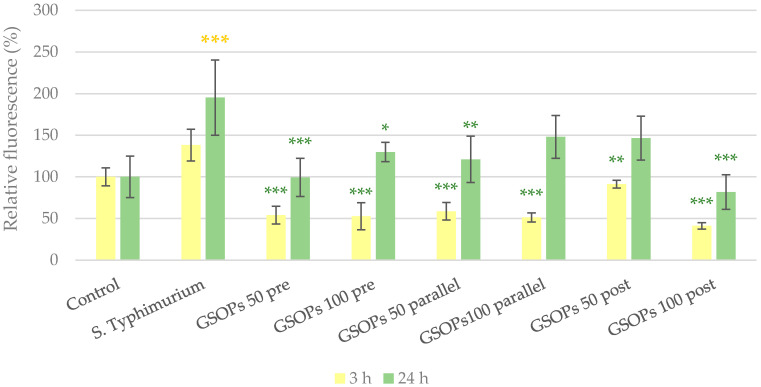
Paracellular permeability of IPEC-J2 cells 3 and 24 h after one hour of treatment with *Salmonella* Typhimurium and grape seed oligomeric proanthocyanidins (GSOPs). Control —treatment with plain medium; *S*. Typhimurium—treatment with 10^6^ CFU/mL *S*. Typhimurium; GSOPs 50, 100 pre—pre-treatment before *S*. Typhimurium infection with 50 and 100 μg/mL GSOPs, respectively; GSOPs 50, 100 parallel—parallel treatment of *S*. Typhimurium infection with 50 and 100 μg/mL GSOPs, respectively; GSOPs 50, 100 post—treatment after *S*. Typhimurium infection with 50 and 100 μg/mL GSOPs, respectively. Data are shown as means with standard deviation and expressed as relative fluorescence, considering the mean value of control as 100%. *N* = 6/group. Significant difference: * *p* < 0.05, ** *p* < 0.01, *** *p* < 0.001, in yellow: compared to the untreated control, in green: compared to *S*. Typhimurium treatment.

In the experiment with *S*. Typhimurium, 24 h after treatment, significantly increased paracellular permeability was observed in cells infected with bacteria. However, GSOPs could prevent barrier integrity impairment in most cases. The results are visible in Figure 7.

### 2.5. Bacterial Adhesion

To determine the potential anti-adhesive effect of GSOPs, the amount of bacteria attached to IPEC-J2 cells was tested with colony-forming unit (CFU) counting. In the experiment, a more pronounced effect of GSOPs was observed in the case of *E. coli* than for *S*. Typhimurium. The addition of GSOPs resulted in a significant reduction (43.62–75.12%) in the amount of *E. coli* adhered to IPEC-J2 cells in all treatment groups except for post-treatment with 50 μg/mL GSOPs. For *Salmonella*, only pre-treatment with GSOPs showed significant anti-adhesion activity, with the bacterial count reduction being over 50%. The results are demonstrated in Table 1.

## 3. Discussion

In vitro models represent an important part of the 3R principle (replacement, reduc-tion, refinement) of animal research when testing substances for either veterinary or human usage. IPEC-J2 cell–bacterium co-cultures are informative in vitro models of GI infections of both humans and pigs that have been used previously for testing the protective effects of probiotics [26,27,28,29]. In this study, for the first time, we infected IPEC-J2 cells with *E. coli* and *S*. Typhimurium in order to evaluate the ability of GSOPs to alleviate damage caused by these bacteria. *E. coli* and *Salmonella* are some of the main zoonotic bacteria that can be transmitted from animals to humans via the food chain [14], leading to the spread of antibiotic resistance. The testing of GSOPs and other natural substances can contribute to finding antibiotic alternatives for the prevention and treatment of bacterial infections and consequently decreasing the usage of antibiotics and the emergence of antimicrobial resistance. This is of utmost importance as the latter is one of the most serious health problems worldwide in human and veterinary medicine alike [30].

PAs from various sources have shown antioxidant activity in different experimental settings [31] and multiple oxidative-stress-related diseases [32]. Their activity against oxidative stress was demonstrated previously both in vitro, in human lens epithelial cells [33], human colorectal adenocarcinoma cells [34], and murine macrophages [35], as well as in vivo, in rat models [36,37] and pigs [38,39,40]. Kovács et al. [9] investigated first the antioxidant effect of GSOPs in IPEC-J2 cells treated with LPS, and they reported a significant reduction in IC ROS levels elevated by bacterial endotoxin. The current study supports previous findings with the demonstration of antioxidant activity of PAs in an in vitro model of gastrointestinal infections. Treatment with GSOPs could significantly alleviate oxidative stress caused by both *E. coli* and *S*. Typhimurium on IPEC-J2 cells.

Similar to their antioxidant property, the anti-inflammatory effect of PAs has also been reported previously. In adenocarcinomic human alveolar basal epithelial cells, PAs could decrease interferon-γ, TNF-α and IL-1β levels induced by treatment with benzo(*a*)pyrene [41]. In human hepatic stellate cells, PA pre-treatment before LPS addition could reduce mRNA expression of IL-1β, IL-6 and IL-8 [42]. In LPS-treated murine macrophages, PAs could also inhibit TNF-α, IL-1β and IL-6 production [43]. Furthermore, in several cell culture models of intestinal dysfunction, established with human colorectal adenocarcinoma cells, PAs from various sources could decrease the level of inflammatory mediators (e.g., TNF-α, IL-6 and IL-8) and had a protective effect on the cell layer integrity [22]. Our findings are in accordance with the reported literature and demonstrate the above-mentioned beneficial effects of PAs in different experimental settings. GSOPs had significant anti-inflammatory activity (by reducing IL-6 and IL-8 levels) and a barrier protective effect in IPEC-J2 cells infected with *E. coli* and *S*. Typhimurium.

The ability of PAs to inhibit bacterial adherence is most extensively described in the case of the efficacy of cranberry-derived PAs in reducing the adhesion of *E. coli* to uroepithelial cells and therefore preventing urinary tract infections [18,44,45,46]. Besides this indication, adherence of *E. coli* to vaginal epithelial cells [47] and buccal epithelial cells [48] could also be decreased by PAs. In terms of other pathogens, PAs possess anti-adhesive effect against *S*. Typhimurium [19], *Proteus mirabilis* [20] and *Candida albicans* [49]. In this study, GSOPs could inhibit the adhesion of *E. coli* and *S*. typhimurium to porcine intestinal epithelial cells, which supplements the above-mentioned data with findings on the IPEC-J2 cell line.

The structure of PAs can have an impact on their antibacterial activity. An anti-adhesive effect against uropathogenic bacteria has been reported about A-type PAs but could not be observed in the case of compounds with a B-type structure [20,46]. However, both A- and B-type PAs could show bacteriostatic and bactericidal effects against pathogens such as *E. coli* and *S*. Typhimurium [9,50,51,52], and PAs with a B-type structure seemed to be more effective in some cases [51,52]. Monomer units from PAs can also possess antibacterial characteristics. Gallic acid, catechin and epicatechin, which are also found in GSOPs, showed bacteriostatic and bactericidal effects against different bacterial strains, including *E. coli* and *S*. Typhimurium [53]. Epicatechin-gallate was effective against *E. coli* as well [54]. Furthermore, gallic acid, epicatechin and epicatechin-gallate showed synergistic or additive effects in combination with different antibiotics [54]. In the current study, GSOPs—containing only B-type linkages between the monomer units [21]—were tested and demonstrated potent anti-adhesive activity against enteropathogenic bacterial strains.

## 4. Materials and Methods

### 4.1. Chemicals and Instruments

GSOPs (Reference Standard of the United States Pharmacopeia; main components: procyanidin B1, procyanidin B2, gallic acid, catechin, epicatechin and epicatechin-3-*O*-gallate; 0.988 mg of purified grape seeds oligomeric proanthocyanidins per mg of material on the anhydrous basis) were obtained from Sigma-Aldrich (Darmstadt, Germany).

Supplier of other chemicals used in this study (growth medium of cells; Neu-tral Red dye; dichloro-dihydro-fluorescein diacetate (DCFH-DA) reagent; enzyme-linked immunosorbent assay (ELISA) kits; fluorescein isothiocyanate–dextran 4 kDa (FD4) dye; Triton X-100) was Sigma-Aldrich (Darmstadt, Germany) as well. ChromoBio Coliform and ChromoBio Salmonella Plus Base selective agars were obtained from Biolab Zrt. (Budapest, Hungary), while cell culture plates were purchased from Corning Inc. (Corning, NY, USA). SpectraMax iD3 (Molecular Devices, San José, CA, USA) was used for absorbance and fluorescence measurement. Statistical analysis of the obtained data was conducted with R software (R Foundation for Statistical Computing, Vienna, Austria).

### 4.2. IPEC-J2 Cell Line

Experiments were performed on the IPEC-J2 cell line that was kindly provided by Dr. Jody Gookin (Department of Clinical Sciences, College of Veterinary Medicine, North Carolina State University, Raleigh, NC, USA). Cells were cultured on 37 °C, with 5% CO_2_, in the 1:1 mixture of Dulbecco’s Modified Eagle’s Medium and Ham’s F-12 Nutrient (DMEM/F12) that contained the following supplementations: fetal bovine serum (5%), in-sulin (5 μg/mL), transferrin (5 μg/mL), selenium (5 ng/mL), epidermal growth factor (5 ng/mL) and penicillin-streptomycin (100-100 U/mL) for cell culturing (full DMEM/F12). Experiments were performed with IPEC-J2 cells at a passage number of approximately 50 and working solutions were prepared with plain DMEM/F12 without supplementation. For the investigations, cells were seeded onto 96- (Neutral Red), 24- (adhesion) or 6-well (DCFH-DA, ELISA) polystyrene cell culture plates, and 12-well polyester membrane inserts (FD4, pore size: 0.4 μm) and were incubated until forming a differentiated, confluent monolayer, which was regularly inspected under light microscope.

### 4.3. Bacterial Strains

*E. coli* and *S*. Typhimurium strains, which originated from GI infections in pigs, were used in the experiments. Bacteria were kept frozen at −80 °C in Micro-bank tubes until the beginning of investigations, when they were propagated in plain DMEM/F12 for 18–24 h at 37 °C, with 5% CO_2_ to mimic culture conditions of IPEC-J2 cells. Concentration of the overnight bacterial suspensions was determined with CFU counting.

### 4.4. Cell Viability Determination

To determine the maximum tolerable concentration of bacteria for co-culturing with IPEC-J2 cells, cell viability assay was performed with different amounts of bacteria. IPEC-J2 cells were cultured in full DMEM/F12 on 96-well microplates until a confluent monolayer was formed. Prior to bacterial infection, the medium was removed, and cells were washed with phosphate-buffered saline (PBS) and incubated in plain DMEM/F12 in order to eliminate antibiotic residues remaining from full DMEM/F12. Both bacterial strains were added to the cells at the concentrations of 10^4^, 10^6^ and 10^8^ CFU/mL that were prepared by dilution with plain medium based on the results of CFU counting. Control cells received only plain medium. Treated and control cells were incubated for 1 h (37 °C, 5% CO_2_). When the supernatants were removed, cells were washed with PBS and then received full DMEM/F12 to prevent bacterial overgrowth. The ratio of living cells was determined 24 h later with the Neutral Red method [55]. Absorbance measurement (on 540 nm) was performed with SpectraMax iD3. Based on the results of the cell viability assay, bacterial suspensions with the concentration of 10^6^ CFU/mL were used in further experiments in the case of both strains. Effect of GSOPs alone on cell viability has been previously tested and they showed no adverse effect up to 200 μg/mL for 24 h [9].

### 4.5. Experimental Design

For all investigations in the co-culture, similar experimental design and treatment groups were used. Cells were cultured in full DMEM/F12 until reaching a confluent monolayer in each well, and then were washed with PBS and incubated in plain DMEM/F12 before all experiments (to remove antibiotic residues). Afterwards, some of them were infected with bacteria at the concentration of 10^6^ CFU/mL without previous, parallel, or sub-sequent GSOP supplementation. Other cells received GSOP treatment (50 and 100 μg/mL) 1 h prior, together, or 1 h after the bacterial infection (10^6^ CFU/mL). Concentrations of GSOPs were determined based on previous investigations [9]. Both GSOP working solutions and bacterial suspensions were prepared with plain DMEM/F12, while control cells received only plain medium. The treatment groups of the experiment are summarized in Table 2. All treatments were applied on cells for 1 h, which was followed by rinsing with PBS and adding antibiotic containing DMEM/F12 on them to prevent bacterial overgrowth in cases when further incubation was necessary.

### 4.6. IC ROS Level Determination (DCFH-DA)

To investigate the potential antioxidant effect of GSOPs in the IPEC-J2–bacterium co-culture, DCFH-DA assay was used. For the assay, cells were cultured on 6-well plates, and the above-described treatments were performed on them, followed by 24 h of incubation in antibiotic-containing (100 U/mL penicillin and 100 U/mL streptomycin) medium. For detecting the amount of IC ROS, 10 µM DCFH-DA dye was used, which can be oxidized to a detectable fluorescent product, di-chloro-fluorescein (DCF) by several IC free radicals, making the assay representative for quantification of overall oxidative stress in the investigated cells [56]. Higher amount of IC ROS leads to more DCF production and consequentially increased fluorescence values. The dye was applied on cells for 1 h, then cells were rinsed, scraped and centrifugated (10 min, 3000 g). After centrifugation, fluorescence of the obtained supernatants was measured with SpectraMax iD3 (excitation wavelength: 485 nm, emission wavelength: 535 nm).

### 4.7. Interleukin Level Determination (ELISA)

To determine the interleukin production of cells affected by bacteria and GSOPs, cells were cultured on 6-plate wells and the previously detailed experimental settings were fol-lowed. Samples were taken from the cell supernatants 6 h after the end of treatments [27,57,58] for IL-6 and IL-8 measurement with porcine-specific IL-6 and IL-8 ELISA kits according to the instructions of the manufacturer. At the end of the protocol, absorbance measurement of the samples was performed with SpectraMax iD3 (on 450 nm). Higher absorbance values indicated an increased amount of interleukins in the samples.

### 4.8. Paracellular Permeability Determination (FD4)

To evaluate effect of bacteria and GSOPs on barrier integrity of the cell layer, IPEC-J2 cells were grown on 12-well membrane inserts for performing the treatments specified in Section 4.5. Afterwards, 0.25 mg/mL FD4 tracer dye was applied on them (i.e., in the apical compartment of wells), and samples were taken 3 and 24 h later from the basolateral compartment (all sampling times measured from the end of treatment). Amount of FD4 in the samples (i.e., ratio of dye that could penetrate through the cell layer) was detected by the fluorescent method with SpectraMax iD3 (excitation wavelength: 485 nm, emission wavelength: 535 nm). Higher fluorescence values indicated increased paracellular permeability as a result of barrier integrity disruption. Transepithelial electrical resistance (TEER) values were measured prior to the experiment to evaluate formation of a confluent, differentiated monolayer.

### 4.9. Bacterial Adhesion Assay

To determine potential anti-adhesive effect of GSOPs, cells cultured on 24-well plates were treated in the above-mentioned manner. After removal of the supernatants (i.e., bacteria not attached to IPEC-J2 cells) and washing with PBS, cells were lysed with 1% Triton X for 30 min on a shaker to release adhered bacteria. A serial dilution was then prepared from the homogenized suspensions in each well and inoculated on selective agar plates (ChromoBio Coliform for *E. coli* and ChromoBio Salmonella Plus for *S*. Typhimurium) for overnight incubation, followed by CFU counting on the next day. All treatments were performed in 4 replicates. Results are presented as the average of replications compared to CFU count of adhered bacteria without GSOPs treatment.

### 4.10. Statistical Analysis

R 3.3.2 (2016) software was used for statistical analysis of data, including the com-parison of mean values of different groups with one-way ANOVA and Tukey post hoc test (significance: *p* < 0.05).

## 5. Conclusions

In this study, for the first time, we demonstrated protective effects of grape seed oligomeric proanthocyanidins in IPEC-J2 cell–bacterium co-culture. GSOPs were able to alleviate oxidative stress, inflammation and barrier integrity impairment caused by *Escherichia coli* and *Salmonella* Typhimurium. Furthermore, GSOPs could significantly reduce the amount of bacteria adhered to IPEC-J2 cells. Based on these findings, GSOPs might be used in the future as antibiotic alternatives for the prevention and treatment of gastrointestinal bacterial infections, but further in vivo studies should be conducted to support their application. The obtained results are not only important for swine health management, but might also be interpreted for human medicine due to properties of the IPEC-J2 cell line and the zoonotic nature of the used bacteria.

## Figures and Tables

**Table 1:** Reduction in the amount of bacteria adhered to IPEC-J2 cells by one hour of treatment with grape seed oligomeric proanthocyanidins (GSOPs). GSOPs 50, 100 pre—pre-treatment before bacterial infection with 50 and 100 μg/mL GSOPs, respectively; GSOPs 50, 100 parallel—parallel treatment of bacterial infection with 50 and 100 μg/mL GSOPs, respectively; GSOPs 50, 100 post—treatment after bacterial infection with 50 and 100 μg/mL GSOPs, respectively.

	*Escherichia coli*		*Salmonella* Typhimurium	
Treatment	Reduction	*p* Value	Reduction	*p* Value
GSOPs 50 pre	−62.35%	*p* < 0.001	−51.14%	*p* < 0.05
GSOPs 100 pre	−75.12%	*p* < 0.001	−57.55%	*p* < 0.05
GSOPs 50 parallel	−43.62%	*p* < 0.05	−24.03%	*p* = 0.16
GSOPs 100 parallel	−44.25%	*p* < 0.01	−30.66%	*p* = 0.11
GSOPs 50 post	−23.27%	*p* = 0.46	−5.38%	*p* = 0.34
GSOPs 100 post	−56.35%	*p* < 0.001	−17.39%	*p* = 0.21

**Table 2:** Treatment groups in the co-culture experiments.

	GSOPs	Bacterium
Control	−	−
*E. coli*	−	*E. coli* 10^6^ CFU/mL
*S*. Typhimurium	−	*S*. Typhimurium 10^6^ CFU/mL
Pre-treatment 50 *E. coli*	50 μg/mL 1 h prior to infection	*E. coli* 10^6^ CFU/mL
Pre-treatment 50 *S*. Typhimurium	50 μg/mL 1 h prior to infection	*S*. Typhimurium 10^6^ CFU/mL
Pre-treatment 100 *E. coli*	100 μg/mL 1 h prior to infection	*E. coli* 10^6^ CFU/mL
Pre-treatment 100 *S*. Typhimurium	100 μg/mL 1 h prior to infection	*S*. Typhimurium 10^6^ CFU/mL
Parallel treatment 50 *E. coli*	50 μg/mL together with infection	*E. coli* 10^6^ CFU/mL
Parallel treatment 50 *S*. Typhimurium	50 μg/mL together with infection	*S*. Typhimurium 10^6^ CFU/mL
Parallel treatment 100 *E. coli*	100 μg/mL together with infection	*E. coli* 10^6^ CFU/mL
Parallel treatment 100 *S*. Typhimurium	100 μg/mL together with infection	*S*. Typhimurium 10^6^ CFU/mL
Post-treatment 50 *E. coli*	50 μg/mL 1 h after infection	*E. coli* 10^6^ CFU/mL
Post-treatment 50 *S*. Typhimurium	50 μg/mL 1 h after infection	*S*. Typhimurium 10^6^ CFU/mL
Post-treatment 100 *E. coli*	100 μg/mL 1 h after infection	*E. coli* 10^6^ CFU/mL
Post-treatment 100 *S*. Typhimurium	100 μg/mL 1 h after infection	*S*. Typhimurium 10^6^ CFU/mL

## Data Availability

All data that support the above-detailed findings can be obtained from the corresponding author upon request.
